# Clinical Evidence on the Potential Beneficial Effects of Diet and Dietary Supplements against COVID-19 Infection Risk and Symptoms’ Severity

**DOI:** 10.3390/medsci12010011

**Published:** 2024-02-17

**Authors:** Eleni Pavlidou, Efthymios Poulios, Sousana K. Papadopoulou, Aristeidis Fasoulas, Antonios Dakanalis, Constantinos Giaginis

**Affiliations:** 1 Department of Food Science and Nutrition, School of Environment, University of the Aegean, 81400 Lemnos, Greece; elen.p.pavl@gmail.com (E.P.); epoulios@aegean.gr (E.P.); athanarist@gmail.com (A.F.); 2 Department of Nutritional Sciences and Dietetics, School of Health Sciences, International Hellenic University, 57400 Thessaloniki, Greece; 3 Department of Mental Health, Fondazione IRCCS San Gerardo dei Tintori, Via G.B. Pergolesi 33, 20900 Monza, MB, Italy; antonios.dakanalis@unimib.it; 4 Department of Medicine and Surgery, University of Milan Bicocca, Via Cadore 38, 20900 Monza, MB, Italy

**Keywords:** COVID-19 pandemic, dietary supplements, dietary patterns, vitamins, trace elements, omega-3 fatty acids, Mediterranean diet, probiotics, infection, virus

## Abstract

Background: Diet and dietary supplements aim to add trace elements, vitamins, and minerals to the body to improve human health and boost the immune system. In the previous few years, the new SARS-CoV-2 coronavirus strain has been threatening the health of individuals and public health more broadly, with rates of intensive care unit cases on the rise, while long-term COVID-19 complications are persisting until today. In the peculiar circumstances of the COVID-19 pandemic, in combination with disease prevention techniques, the strengthening of the immune system is considered particularly important to enable it to effectively respond to and eliminate the SARS-CoV-2 viral pathogen in the event of infection. The purpose of the current literature review is to thoroughly summarize and critically analyze the current clinical data concerning the potential beneficial effects of diet and dietary supplements against COVID-19 infection risk and symptoms’ severity. The micronutrients/supplements examined in this study in relation to COVID-19 infection are vitamins A, B, C, and D, zinc, selenium, magnesium, iron, omega-3 fatty acids, glutamine, resveratrol, beta-glucans, and probiotics. The potential effects of dietary patterns such as the Mediterranean diet against SARS-CoV-2 infection risk and symptoms’ severity were also analyzed. Our literature review suggests that micro- and macronutrient supplementation and a healthy diet and lifestyle may provide support to immune system function, with beneficial effects both before and during SARS-CoV-2 infection. However, additional studies are recommended to draw safe conclusions and formulate dietary recommendations concerning dietary supplements and their possible effects on preventing and co-treating COVID-19 disease.

## 1. Introduction

Global health, economic, and psychological upheavals have been caused by the COVID-19 pandemic, which began in China in the last month of 2019. To prevent the disease from spreading, almost all countries were compelled to authorize rigorous health protocols and social isolation strategies. Specifically, in countries that experienced prolonged confinement actions, such as social isolation orders, teleworking [1], and the shutdown of schools and companies, these measures led to social isolation, which exerted a substantial negative effect on people’s mental health [2] and everyday habits. The immunological system’s action and the possibility of its relationship with nutrition received particular interest in an attempt to reduce infection in order to promote an improved functional response and an elevated, applicable defense against SARS-CoV-2 [3,4]. Sufficient food consumption seems to promote a quite effective defense against the virus and assists in controlling the infection in the outcomes of disease [5]. It should be noted that the immunological system is activated to a greater extent in the case of an organism which is subjected to an infective factor [6]. The above enhanced activity is accompanied by an increased metabolism rate that needs supplementary energy, biosynthesis compounds, and controlling substances from food [7].

In view of the above considerations, adopting healthy nutritional habits could prove helpful in reinforcing the immunological system, which is important for the treatment of diverse infections. On the contrary, a lack of healthy nutritional habits has adverse effects related to inflammation and oxidative stress, and thus may influence the disease outcome by deteriorating the immunological system [7]. Although COVID-19 infection and its related symptoms were initially supposed to be acute in nature, several patients have documented permanent and recurring symptoms beyond the COVID-19 infection period [8]. This severe issue has emerged as a novel epidemic termed “long-COVID”, or post-acute sequelae of coronavirus disease (PASC), which has considerably and negatively affected the daily life of persons worldwide [8]. PASC constitutes a condition during which patients continue to experience COVID-19 symptoms, which continue at least three months subsequent to the primary COVID-19 infection [9]. It is estimated that about one-third of COVID-19-infected patients suffered from symptoms like fatigue, muscle aches and pains, and cognitive complications for at least three months post-infection [10].

In this aspect, potential nutritional interventions, in combination with drug treatment, could be considered as an effective strategy to prevent or even treat COVID-19 infection and its associated symptoms. Promising evidence from direct research on COVID-19 and PASC, adjacent to indirect research from analogous respiratory virus infections and post-infection symptoms, has supported the possible impacts of dietary habits in clinical outcomes and symptomatology monitoring [11]. Regarding these emergent considerations, the current literature review aims to thoroughly summarize and scrutinize the current clinical data concerning the possible positive impacts of diet and dietary supplements against COVID-19 infection risk and symptoms’ severity.

### 1.1. Dietary Supplements

Food supplements can act as adjuvants and may contribute to supplementing the normal diet without being a substitute for food. They are concentrated sources of nutrients marketed in various dosage forms (capsules, lozenges, tablets, pills, powder sachets, sachets of liquid products, vials of liquid products, dropper bottles, etc.). Dietary supplements do not require FDA approval for their safety and efficacy because they are not therapeutic drugs but only essential components for promoting the protection of immunological, metabolic, physical, and mental development [12]. Diverse supplements and nutrients, which were investigated for their beneficial effects on viral infections [13,14,15,16] have sparked the interest of the scientific community in an effort to adopt preventive and effective measures and treatments against COVID-19 [4].

There are also several types of herbal supplements/natural products which have been considered as they may act against diverse infections, including COVID-19 [17]. The most widely used are ginger, garlic, honey, turmeric (curcumin), lemon, black seed, cinnamon, and anise. However, there are not sufficient research data thus far suggesting that they could provide any benefit against COVID-19 infection and disease symptoms’ severity [17]. A previous systematic review identified 12 randomized controlled trials examining diverse herbal medicine interventions on individuals suffering from COVID-19 [18]. Although some of the included studies provided preliminary evidence that herbal supplements may contribute to the recovery of COVID-19 patients, most of them suffered from severe methodological biases [18]. Thus, they should be taken into careful consideration because of their overall low quality, highlighting the strong demand to perform more well-organized clinical trials.

### 1.2. SARS-CoV-2

The World Health Organization (WHO) received a report of an unexplained pneumonia outbreak at the end of 2019 in Wuhan, China. Following virus isolation from bronchoalveolar lavage fluid samples from individuals with pneumonia symptoms, the viral RNA sequence was determined, and this infectious agent was identified as beta-coronavirus, a previously unknown coronavirus type [19]. In January 2020, the WHO recognized an increased incidence of serious acute respiratory syndrome triggered by coronavirus 2 as an epidemic condition. The International Committee for the Classification of Viruses identified SARS-CoV-2 as the causal factor; however, in February 2020, the WHO reclassified this disorder as COVID-19 (ICTV) [20].

SARS-CoV-2 constitutes the third consecutive zoonotic human respiratory coronavirus of the twenty-first century to damage the respiratory system and represent a serious hazard for public health, next to the introduction of SARS-CoV in 2002 and MERS-CoV in 2012, respectively. To maintain normal function and improve the immune system, it is essential to consume sufficient vitamins and minerals, especially when dealing with disorders such as COVID-19 infection [21].

## 2. Methods

**Figure 1:**
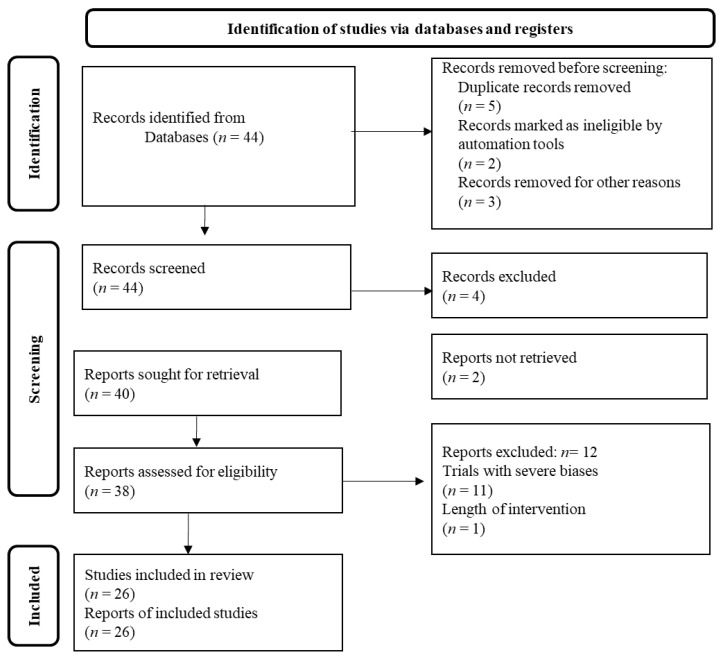
Prisma flow chart diagram.

This is a thorough review of the currently available clinical data on the potential beneficial effects of diet patterns and dietary supplements against the COVID-19 pandemic. Specifically, the most accurate scientific databases, e.g., PubMed, Scopus, Web of Science, and Google Scholar were thoroughly examined using relevant keywords like “COVID-19 pandemic”, “virus”, “dietary supplements”, “dietary patterns”, “vitamins” “trace elements”, “bioactive compounds”, “probiotics”, “polyphenols”, etc., to retrieve the currently available clinical studies in humans. Inclusion criteria were any longitudinal, cross-sectional, descriptive, prospective, preliminary, and case-report clinical studies performed on humans. Exclusion criteria were all studies written in a language other than English. Reviews, comments’ reports, editorials, letters to the editor, and abstracts in conference proceedings were not included in the final analysis. The retrieved findings were screened based on their relevance and the most validated of them were chosen and mentioned underneath based on the Prisma flow chart diagram illustrated in Figure 1. The recovered clinical studies were additionally comprehensively checked for potential similar studies reported in their manuscript.

## 3. Results

### 3.1. SARS-CoV-2 and Dietary Supplements

**Figure 2:**
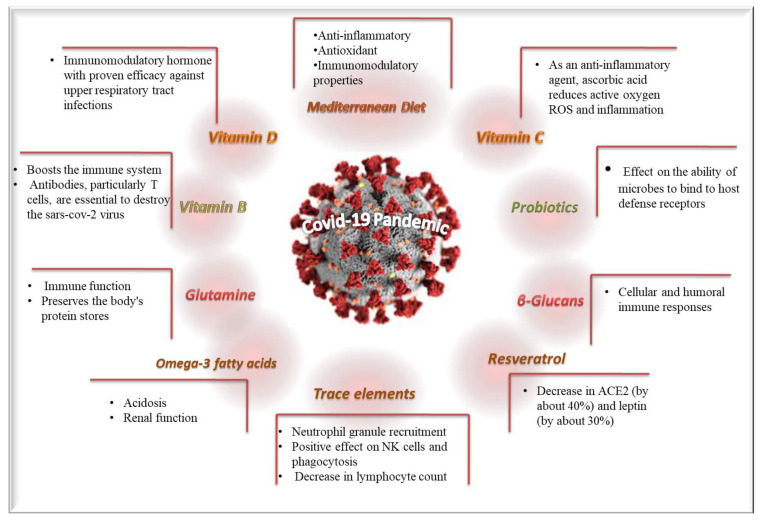
The most important beneficial effects and the underlining mechanisms of diet and dietary supplements against COVID-19.

Diverse clinical studies have been conducted to examine the potential beneficial effects of diet patterns and dietary supplements against the COVID-19 pandemic. The most important beneficial effects and the underlining mechanisms of diet patterns and dietary supplements are depicted in Figure 2. Moreover, the currently available retrieved clinical studies with adequate methodological validity concerning the possible impacts of dietary supplements/patterns against COVID-19 infection and disease’ symptoms severity are included in Table 1.

#### 3.1.1. Trace Elements

Zinc (Zn) constitutes a trace element having a recommended daily intake of 15 mg, which participates in the innate and developed response to viral infections [22]. Zn is involved in neutrophil granule recruitment and can exert a beneficial impact on NK cells and phagocytosis [23]. Notably, Zn deficiency is associated with a decrease in lymphocyte count. Zn supplementation also raises the amount of T and NK cells, as well as enhancing interleukin (IL)-2 expression. With respect to SARS-CoV-2, it was found to suppress the biosynthesis, reproduction, and transcription system of coronaviruses. Zn supplementation additionally appears to suppress symptoms associated with lower respiratory tract infection (as in the case of COVID-19) [23]. In this context, a recent clinical study conducted on 88 COVID-19-positive children patients and 88 healthy children showed that the Zn mean concentrations of the COVID-19 positive children were substantially decreased compared to the healthy children [24]. In addition, a case-control clinical study including 93 adult patients suffering from COVID-19 and 186 healthy adults with no COVID-19 symptoms also revealed that serum Zn concentrations were decreased in COVID-19-positive adults than the healthy individuals [25]. However, it should be emphasized that Zn supplementation above the recommended doses may exert harmful side effects like copper deficiency and medication absorption decrease [26]. Thus, it remains uncertain whether Zn supplementation could be safely and effectively used against COVID-19 infection. In agreement with the previous studies, a systematic review by Balboni et al. identified only a small number of surveys examining the potential impacts of Zn supplements against COVID-19 pathology, which did not verify efficacy [27]. Notably, this systematic review found 22 unpublished forthcoming clinical trials, 19 on Zn, 1 on selenium (Se), and 2 for both of them. Thus, Balboni et al. concluded that although the preventive or co-therapeutic Zn or Se supplementation interventions against COVID-19 are at present unjustified, the findings of the ongoing studies will be published, and this could alter the currently existing conclusions [27].

Selenium (Se) with a recommended daily intake of 35 μg constitutes an essential trace element, which exerts a crucial impact as a redox agent since it occurs in the form of selenocysteine in the catalytic centers of many selenoproteins. The synthesis of selenocysteine requires a continuous resource of the amino acid serine. Se deficiency potentially affects both forms of the immunological response concerning viruses’ pathogenesis [28].

Remarkably, oxidative stress together with Se deficiency works synergistically in converting the virus from mild to highly infectious. In this aspect, Se as an antioxidant agent plays a crucial role as a cofactor in a family of enzymes which functions adjunctively with vitamin E to decrease reactive oxygen species known as ROS. An impaired antioxidant defense may herald an excessive inflammation response to the host similar to non-active infection [28]. The most potent antioxidant Se-dependent enzymes include glutathione peroxidases (GPXs) and thioredoxin reductases (TXNRDs) that require Se to be functional. Other selenoproteins such as selenoprotein K (SELENOK) and selenoprotein S (SELENOS) are related to immunoregulation. Another factor that cooperates with these seleno-dependent enzymes is the cofactor Q10. This supplement appeared to reduce the specific inflammation response, especially concerning infections such as COVID-19 that exhibit hyperactive inflammation; however, there is no clinical study thus far for such an action of cofactor Q10 [28].

A population-based, retrospective study found a relation of the cure COVID-19 rates with Se status [29]. However, this study had several limitations preventing sufficient conclusions from being drawn [29]. In this aspect, a critical review study reported that Se levels may affect the human response to SARS-CoV-2 pathology. Moreover, Se status could be one among the various risk factors that could affect the outcome of SARS-CoV-2 disease, mainly in individuals with low or suboptimal Se consumption [30]. However, at this time, there is no substantial study to support whether Se status may be related with COVID-19 pathology.

Magnesium (Mg) with a recommended daily intake of 350 mg constitutes an important essential trace element and the main cation of human cells localized in mitochondria. Mg has already been used in lung diseases characterized by inflammation such as asthma and pneumonia. Specific studies have demonstrated that Mg sulphate enhanced the bronchodilation induced by an administered β2 agonist in individuals with a chronic obstructive lung disorder [31]. Patients with COVID-19 experienced respiratory tract symptomatology, most notably cough, sore throat, nasal congestion, and dyspnea. Mg sulfate can act as a calcium antagonist that usually finds application in suppressing bronchial smooth muscle fiber reduction and stimulating bronchodilation [31].

In this aspect, hypomagnesemia and hypermagnesemia might have major negative consequences, perhaps by progressing from a moderate to a severe development of COVID-19. Subjects presenting reduced Mg concentrations could be more prone to develop and enhance this illness. There is insufficient evidence thus far concerning the Mg status of COVID-19 patients presenting varying intensity of symptoms’ severity [30]. In this context, a cross-sectional retrospective survey on 1064 individuals with COVID-19 staying in a hospital showed that a Mg-to-Ca ratio ≤ 0.20 was highly associated with death rates in individuals with progressed COVID-19 disease [32].

Iron (Fe) with a recommended daily intake of 15 mg is a common mineral, which plays a crucial role for the enzymes’ activity of immune cells, like neutrophils and lymphocytes, while serious Fe shortage has been related to a high risk of diverse infections [33]. On the contrary, excessive Fe levels have been found to raise the probability of several infections [34]. In this aspect, it should be noted that COVID-19 has been shown to affect Fe homeostasis, while a pilot, retrospective cohort survey in 50 individuals with COVID-19 staying in a hospital showed a relation between decreased serum Fe concentrations and COVID-19 related death rates and symptoms’ intensity [35,36]. This is a first clinical study, which highlights the strong demand to perform future well-designed clinical studies on this topic.

**Table 1:** The currently available clinical studies with adequate methodological validity concerning the possible impacts of dietary supplements/patterns against COVID-19 infection and disease symptoms’ severity.

Dietary Supplements/Patterns	Study Design	Main Findings	Ref.
Zn Control group: 84.86 ± 19.65 μg/dL, Patients group: 70.32 ± 15.94 μg/dL	Prospective cohort study 88 COVID-19-positive pediatric patients and 88 healthy children.	Zn mean concentrations of COVID-19 positive children were significantly decreased compared to the healthy children.	Doğan et al., 2022 [24]
Vitamin D Control group: 18.14 ± 8.81 ng/mL, Patients group: 11.73 ± 4.7 ng/mL	Prospective cohort study 88 COVID-19 positive pediatric patients and 88 healthy children.	The mean serum vitamin D levels of COVID-19 positive patients were statistically significantly lower than the control group. Severe serum vitamin D deficiency in COVID-19-positive patients was statistically significantly higher than in the control group.	Doğan et al., 2022 [24]
Zn Control group: 86.66 ± 11.76 μg/dL, Patients group: 67.61 ± 15.10 μg/dL	Case-control study 93 hospitalized patients with COVID-19 and 186 healthy subjects with no symptoms of COVID-19.	Serum Zn concentrations were reduced in individuals with COVID-19 compared to healthy ones.	Elham et al., 2021 [25]
Ca (Control group: 9.50 ± 0.52 mg/dL, Patients group: 9.46 ± 0.58 mg/dL)	Case-control study 93 hospitalized patients with COVID-19 and 186; healthy subjects with no symptoms of COVID-19.	The serum calcium level significantly differed between patients and healthy groups among men and women.	Elham et al., 2021 [25]
Vitamin D Control group 22.83 ± 12.97 ng/mL, Patients groups: 27.50 ± 15.35 ng/mL)	Case-control study 93 hospitalized patients with COVID-19 and 186 healthy subjects with no symptoms of COVID-19.	Serum vitamin D levels in COVID-19 patients are lower than in the control group.	Elham et al., 2021 [25]
Zn 50 mg for 10 days	Randomized clinical open-label trial Standard care patients: *n* = 50; Interventional group: *n* = 58.	Zn supplementation was not significantly related with reduced COVID-19 symptomatology intensity, as well as duration compared with standard care.	Thomas et al., 2021 [26]
Vitamin C Control patients: standard care Interventional group: 8000 mg for 10 days	Randomized clinical open-label trial Standard care patients: *n* = 50; Interventional group: *n* = 48.	Vitamin C supplementation was not significantly related with reduced COVID-19 symptomatology intensity, as well as duration compared with standard care.	Thomas et al., 2021 [26]
Se	Population-based, retrospective study	COVID-19 cure rates were associated with Se levels.	Zhang et al., 2020 [29]
Mg-Ca ratio cut-off: 0.20	Cross-sectional retrospective study Recovery patients: *n* = 510; Death patients: *n* = 554.	A Mg-to-Ca ratio ≤ 0.20 was highly associated with death rates in individuals presenting severe COVID-19.	Guerrero-Romero et al., 2022 [31]
Vitamin A Control group: hydroxychloroquine Experimental group: hydroxychloroquine + 25,000 IU/d oral vitamin A for 10 days	Triple-blind controlled randomized clinical trial Control group: *n* = 91; Experimental group: *n* = 91.	There were significant differences in reduction in fever, body pain, exhaustion, and white blood cell count for the intervention group compared to control group.	Rohani et al., 2022 [32]
Fe Outpatients group = 8.6 (5.0–14.9) μmol/L Inpatients group = 2.6 (1.8–3.9) μmol/L Outpatients admitted to hospital because of health deterioration = 3.2 (2.4–4.6) μmol/L	Small, retrospective cohort study 204 outpatients; 81 inpatients; 23 outpatients later admitted to hospital because of health deterioration.	An association between low serum Fe levels with COVID-19 related mortality and disease severity was noted. Serum iron and ferritin were significantly associated with hospitalization, whereby doubling of serum iron was associated with a 6.7-fold lower odds of hospitalization.	Hippchen et al., 2020 [36]
Folic acid (Vitamin B9), 5 mg or greater	Randomized, double-blind, placebo-controlled trial	No impact on disease progression was observed.	Wiltshire et al., 2020 [37]
Vitamin C	Retrospective cohort study Participants: *n* = 372 720 UK, *n* = 45 757 Sweden *n* = 27 373.	Vitamin C supplementation did not exert any significant effect against SARS-CoV-2 infection. There was only a positive association in male participants aged >60 years, receiving vitamin C supplements for testing positive for SARS-CoV-2.	Louca et al., 2021 [38]
Vitamin C Enteral vitamin C in a dose of 1000 mg daily with a median duration of administration of 11 days	Two-center, non-interventional, retrospective cohort study Control group: *n* = 581 patients; Interventional group: *n* = 158 patients.	Vitamin C as an adjunctive therapeutic agent against COVID-19 was not related with mortality benefits; however, it was associated with a reduced prevalence of thrombosis.	Al Sulaiman et al., 2021 [39]
Vitamin D Control group: 100,000 IU. Intervention group: 400,000 IU Duration: 28 days	Open-label, multicenter, randomized controlled superiority clinical trial Control group: 130 patients; Intervention group: 130 patients.	Enhanced vitamin D supplement seems to be an effective, well-tolerated, and easily and immediately accessible treatment for COVID-19.	Annweiler et al., 2020 [40]
Vitamin D Standard treatment: 1000 UI oral vitamin D3 Case treatment: 5000 IU oral vitamin D3 Duration: 2 weeks	Multicenter randomized clinical trial Standard control: *n* = 33 hospitalized patients; Case control, *n* = 36 hospitalized patients.	A 5000 IU daily oral vitamin D3 supplementation for 2 weeks reduces the time to recovery for cough and gustatory sensory loss among patients with sub-optimal vitamin D status and mild to moderate COVID-19 symptoms.	Sabico et al., 2021 [41]
Vitamin D Daily oral 1000IU dose of vitamin D3, 150 mg of Mg, and 500 μg vitamin B12 for ≤14 days	Retrospective observational cohort study Control group: *n* = 17 patients; Interventional group: *n* = 26 patients.	A vitamin D/Mg/vitamin B12 combination was associated with a considerable decrease in the percentage of patients with clinical weakening, requiring oxygen support, intensive care support, or both of them.	Tan et al., 2020 [42]
Vitamin D Group A: 25(OH)D levels ˂ 20 ng/mL, *n* = 309 Group B: 25(OH)D levels ± 20 ng/mL, *n* = 155	Multicenter observational study Included patients: *n* = 464.	Serum 25(OH)D levels < 12 ng/mL were strongly associated with COVID-19 severity and mortality among a sample of affected people.	Al Safar et al., [43]
Vitamin D Higher-dose: 3200 IU/day Lower-dose: 800 IU/day Follow-up: 2 weeks	Randomized controlled trial Lower intake group: *n* = 1550; Higher intake group: *n* = 1550.	Vitamin D did not affect the defensive efficacy or immunogenicity of SARS-CoV-2 vaccination when administered to adults presenting lower levels of vitamin D at disease onset.	Jollife et al., 2022 [44]
Vitamin D Group 1: 52,000 IU monthly Group 2: dietary-hygienic measures Follow-up: 3- to 6-months	Randomized controlled clinical trial Hospital workers with 25(OH)D3 levels between 20 and 100 ng/mL and no previous SARS-CoV-2 infection: *n* = 198.	Vitamin D supplement in participants presenting 25(OH)D_3_ concentrations at a range of 20–100 ng/mL exhibited a decreased occurrence of SARS-CoV-2 infection compared to the utilization of nutritional-hygienic measures at a 6-month follow-up.	Romero-Ibarguengoitia et al., 2023 [45]
Omega-3 fatty acids Control group: Hydroxychloroquine Intervention group: Hydroxychloroquine plus 2 grams of Docosahexaenoic acid [DHA] + Eicosapen-taenoic acid [EPA]) Duration: 2 weeks	Single-blind randomized controlled study Control group: *n* = 15; Intervention group: *n* = 15.	Omega-3-supplemented COVID-19 patients showed improved clinical complaints except for bodily pain and tiredness, for appetite, and olfactory. Both CRP and ESR were also reduced by omega-3 supplement than the control group following therapy.	Sedighiyan et al., 2021 [46]
Omega-3 fatty acids Intervention: fortified formula with n3-PUFA for 2 weeks	Double-blind, randomized clinical trial Control group: *n* = 86; Intervention group: *n* = 42.	The intervention group had a significantly enhanced one-month survival rate, arterial pH, HCO_3_, and Be levels, as well as arterial BUN, Cr, and K levels, compared with the control group.	Doaei et al., 2021 [47]
Glutamine Case group consumed 10 g of glutamine supplement three times per day for a duration of 5 days	Case-control clinical study Control group: *n* = 230; Case group: *n* = 232.	Glutamine supplementation significantly increased patients’ appetite relative to the control group, and considerably decreased serum concentrations of TNF-a, high-sensitivity CRP, and IL-1.	Mohajeri et al., 2021 [48]
Resveratrol 30 days of 150 mg/day trans-resveratrol	Placebo-controlled cross-over clinical study Control group: *n* = 10; Intervention group: *n* = 11.	Resveratrol treatment reduced ACE2 in AT, which may inhibit the spread of SARS-CoV-2 in COVID-19.	De Ligt et al., 2022 [49]
Beta-glycan 516.67 mg of β-1,3/1,6-glucan for 30- 35 days	Single-center, randomized, double-blind, placebo-controlled study Control group: *n* = 34; Intervention group: *n* = 33.	A supplement including beta-glycan showed that COVID-19 patients group experienced higher increases in IgG and IgM than the placebo group. The beta-glycan supplement increased the ability to stimulate trained immunity.	Rodriguez et al., 2021 [50]
Probiotics Bifidobacterium strains (25 billion CFUs per capsule), galactooligosaccharides, xylooligosaccharide, and resistant dextrin Treatment for 4 weeks Follow-up for 9 months	Longitudinal fecal metagenomic profiling study Control group: *n* = 10; Intervention group, *n* = 22.	Probiotics dramatically lowered the ARGs reservoir in the intestinal microbiome of individuals with COVID-19 infection.	Su et al., 2022 [51]
Probiotics 4 capsules ImmunoSEB (500 mg/capsule) + ProbioSEB CSC3 (5 billion CFUs/capsule) for 14 days	Randomized, multicentric, double blind, placebo-controlled clinical trial Control group: *n* = 100; Intervention group: *n* = 100.	Probiotics’ supplement treatment resulted in the resolution of fatigue in a higher proportion of patients of the interventional group compared to the control group. Patients in the interventional group had a significantly elevated decrease in total, physical, and mental fatigue scoring at all time points compared to the non-interventional group.	Rathi et al., 2021 [52]
Mediterranean diet adherence Medi-lite adherence score	Observational retrospective study No COVID-19 infected: *n* = 752; COVID-19 infected: *n* = 148.	Patients presenting SARS-CoV-2 infection had a considerably reduced MD compliance (e.g., decreased intake of fruits, vegetables, cereals, and olive oil). Patients presenting SARS-CoV-2 infection with no symptoms documented a decreased consumption of saturated fats compared to those with symptoms. Patients needing hospitalization stated more unhealthy nutritional behaviors compared to both asymptomatic and symptomatic individuals.	Ponzo et al., 2013 [53]
Mediterranean diet adherence Mediterranean diet score (MDS)	Prospective and multipurpose cohort study *n* = 9413 participants; *n* = 369 participants infected by COVID-19.	Individuals with intermediate MD adherence had significantly decreased probability of developing COVID-19.	Perez-Araluce et al., 2022 [54]
Mediterranean diet adherence MedDietScore	Cross-sectional clinical study *n* = 3721 participants.	Higher MD compliance was independently related with a decreased probability of abdominal obesity, enhanced physical activity, higher incidence of good sleep quality, improved quality of life, and reduced probability of anxiety and depression throughout the COVID-19 pandemic period.	Pavlidou et al., 2021 [55]

Zn: zinc, Se: selenium, Mg: magnesium, Ca: calcium, Fe: iron, CRP: c-reactive protein, ESR: erythrocyte sedimentation rate, BUN: blood urea nitrogen, Cr: creatinine, K: potassium, Be: base excess, TNF-a: tumor necrosis factor-a, IL-1: interleukin, ACE2: angiotensin converting enzyme-2, AT: adipose tissue, ARGs: antimicrobial resistance genes, MD: Mediterranean diet.

#### 3.1.2. Vitamin A

Vitamin A has a recommended daily intake of 600 μg and it is essential for the consistent immunity, exerting a crucial impact in the growing of T cells, T helper cells, and B cells. In this aspect, a study by Rohani et al. investigated the role of vitamin A intake on the therapeutic effect of patients presenting COVID-19 [32]. Both groups under study received the standard anti-COVID-19 drug therapy, and one of the groups orally received 25,000 IU/d of vitamin A for 10 days. Clinical symptoms in the two groups did not differ significantly before the intervention. However, there were significant differences in reduction in fever, body pain, exhaustion, and white blood cell count for the experimental group [32]. Even if there is not any other study thus far, this first study seems promising and should trigger the performance of clinical studies in the future, which could reveal positive impacts of vitamin A against COVID-19 risk infection and/or symptoms’ disease severity.

#### 3.1.3. Vitamins B Complex

The normal function of the immunological system and energy metabolism, as well as cell function, all depend on the vitamin B complex [56]. A lack of the vitamin B complex may cause the cytokine levels which reinforce immune system function, particularly innate and adaptive immunological responses, to decline [56]. Its connection to SARS-CoV-2 viral infection and the possibility of utilizing it as a dietary supplement should therefore be evaluated [57].

Cardiovascular issues, neuroinflammation, increases in inflammation, and aberrant antibody responses might be considered as consequences of thiamine deficiency. Thiamine (vitamin B1, recommended daily intake: 1.4 g) shortage could result in insufficient antibody responses, which may subsequently exacerbate disease symptoms [56,58]. This is ascribed to the fact that antibodies, particularly T cells, are essential to destroy the SARS-CoV-2 virus. In addition, thiamine acts as a carbonic anhydrase isoenzyme suppressor. Consequently, it is anticipated that adequate thiamine levels will maintain healthy immunological responses during SARS-CoV-2 infection, and that substantial dosages of thiamine administered to patients during the initial steps of COVID-19 infection could minimize hypoxia and stays in the hospital [56].

When riboflavin (vitamin B2, recommended daily intake: 1.6 mg) and ultraviolet (UV) light are mixed, irreparable damage is caused to nucleic acids like DNA and RNA, inhibiting the reproduction of microorganisms. Both riboflavin and UV light have demonstrated adequate efficacy against MERS-CoV, proposing that they may additionally be beneficial against SARS-CoV-2. In a study conducted by Ragan et al., plasma and blood units that had previously been contaminated with SARS-CoV-2 were subjected to treatment with riboflavin and UV radiation [59]. The infection load of SARS-CoV-2 dropped below the detection limit with a mean viral load reduction of more than 4.79 ± 0.15 logs in plasma and 3.30 ± 0.26 logs in whole blood units. These findings suggested that the use of riboflavin and UV radiation may partially lower the probability of COVID-19 virus transmission via transfusion and may also decrease the number of viral cells in COVID-19 patients who are critically unwell [59]. Even if there is not any clinical study thus far to support the above actions of vitamin B2, this hypothesis seems to have an increased probability of being confirmed in the near future by the performance of well-designed randomized clinical trials.

The B3 vitamin, niacin, with a recommended daily intake of 18 mg, serves as a precursor to nicotinamide adenine dinucleotide (NAD)^+^ and NADP^+^, both of which are essential during systemic chronic inflammation [60]. When NAD^+^ levels are increased, a variety of pathophysiological disorders can be treated. NAD^+^ functions as a co-enzyme in diverse metabolism pathways. Niacin (B3) supplementation may also assist COVID-19 patients in reducing the inflammatory process, which is often triggered by IL-6 and is generated during the early stages of inflammatory cascade [60]. NAD^+^ is known to suppress pro-inflammatory cytokines, IL-1, IL-6, and TNF-α. Moreover, the B3 vitamin decreases neutrophil infiltration and has anti-inflammation benefits in individuals presenting ventilator-induced lung damage in intensive health care units. Niacin also appears to protect the lungs and enhance immune function; hence, it may be utilized as a supplementary agent to the existing therapies for COVID-19 patients [60].

Folic acid is a water-soluble vitamin with a recommended daily intake of 400 μg that is classified as part of the vitamin B complex (vitamin B9). Folic acid intake is essential for human health due to its preventative effects against megaloblastic anemia and neural tube birth defects, as well as other chronic diseases. Folic acid may be recommended as an adjuvant therapy for COVID-19 and early-stage respiratory disorder due to the fact that tetrahydrofolic acid and 5-methyl tetrahydrofolic acid exhibit considerable and constant binding affinities against SARS-CoV-2 [60]. Notably, it has been postulated that elevated dosages of folic acid, 5 mg or higher, could be considered an effective therapeutic approach for pulmonary hypertension and/or refractory severe hypoxemia in individuals presenting serious COVID-19-related pneumonia in which NO treatment is applied [37]. In this context, Ibrahimagić et al. have further supported the above hypothesis, reporting that hyperhomocysteinemia could appear in individuals presenting pulmonary hypertension and the concentrations of folic acid are adversely related with those of homocysteine [61]. However, there is no clinical trial to test this hypothesis until the results of the above clinical trial to be published.

The most bioavailable and active form of vitamin B12, methylcobalamin (recommended daily intake: 6 μg), has been shown by Narayanan and Nair during 2020 [62] to exert the potential to lessen organ damage and COVID-19-related symptoms when administered. Positively polarized single-stranded RNA makes up the SARS-CoV-2 viral genome. One of the proteins encoded by the genome is the NSP12 protein, which is essentially in charge of replicating the viral genome. A homology model of the protein has been used to show that methylcobalamin can bind to the NSP12 protein’s active site [62]. This vitamin has a great affinity for the region of the viral nucleotide bonds that are being received. Additionally, viral replication is impeded because the vitamin’s binding site coincides with those of the entering nucleotide, as well as the RNA substrate [62].

Vitamin B12 exerts a crucial role in promoting red blood cell generation, central nervous system health, myelin formation, cell proliferation, and prompt DNA biosynthesis. Deficiency of vitamin B12 can result in intestinal inflammation, increases in ROS, and oxidative stress. Vitamin B12 can also modulate the intestinal flora. SARS-CoV-2 may interfere with the metabolism of vitamin B12, hence hindering the growth of intestinal flora [60]. Therefore, it is conceivable that COVID-19 infection symptoms like enhanced oxidative stress and lactate dehydrogenase, hyperhomocysteinemia, activation of the coagulation cascade, vasoconstriction, and renal and respiratory vasculopathy, are mainly caused by vitamin B12 deficiency. Additionally, a B12 shortage can result in problems with the digestive, respiratory, and brain systems. Because there is a lack of gastric acid or the intrinsic factor required for active B12 absorption, which makes it difficult for them to absorb this vitamin from diet, the elderly may also have the highest vitamin B12 deficit. This condition may contribute to the elderly’s higher sensitivity to COVID-19 infection [60].

At this time, an ongoing multicenter, double-blind, randomized, and placebo-controlled phase-IIIb trial investigates the treatment of individuals presenting post-COVID-19-syndrome (PC19S) with prednisolone and/or vitamins B1, B6, and B12 for a duration of 28 days in primary care and it is expected to announce its results in the next few months [63]. This clinical trial assesses the viability, protection, and efficiency of treating patients in primary care with prednisolone and/or vitamin B1, B6, and B12 and it will be positive if the advantage of any therapy is proved beyond placebo operationalized via an enhancement of at least three points on the Patient Reported Outcomes Measurement Information System (PROMIS) total score (t-score) [63].

#### 3.1.4. Vitamin C

Vitamin C, also well-recognized as ascorbic acid with a recommended daily intake of 75 mg, is a water-soluble vitamin with strong anti-oxidant properties. Vitamin C is unstable and sensitive to heat and air and is destroyed by oxidation. Vitamin C is also oxidized by smoking, so for smokers the proposed daily consumption of vitamin C is significantly greater than for non-smokers. Vitamin C is found in abundance in green leafy vegetables, tomatoes, peppers, citrus fruits, kiwi fruit, and pineapple, as well as in animal organs such as kidneys and liver. Vitamin C has a wealth of immunomodulatory, anti-inflammatory, antioxidant, anti-thrombotic, and antiviral properties. Ascorbic acid also appears to be involved in T-lymphocyte maturation, as well as phagocytosis and chemotaxis functions [64]. Phagocytes import vitamin C in its oxidized form (dehydroascorbic acid) and transform it into vitamin C (ascorbic acid) and this explains the impact of this vitamin as an anti-oxidant agent. Concerning COVID-19, vitamin C is related to the decrease in several cytokines, so it exerts a key impact in protecting the endothelium from oxidative damage [64].

As an anti-inflammatory agent, ascorbic acid reduces active ROS (such as peroxides, oxygen free radicals, hydroxyl radicals, etc.) and inflammation. As a result, ascorbic acid can stop the lungs from being harmed by ROS. A cytokine storm is specifically brought on by COVID-19 infection, which also causes systemic lung inflammation. Nitric oxide (NO) and other inflammatory mediators, including IL-6 and -CSF, are produced by highly activated macrophages as a result of the infection [64]. As an immunomodulator, ascorbic acid inhibits the motivation of the nuclear factor (NF)-κβ that plays a central role in overall immunity. An elevated dosage of vitamin C promotes the proliferation of T and B leukocytes and nuclear killer (NK) cells. This strengthening of immune system cells suppresses the cytokine storm and improves host immunity as a whole [64].

Furthermore, although SARS-CoV-2 has been shown to reduce the expression of type 1 interferons (IFs), which are indeed the first line of defense of the body against viral infections, ascorbic acid positively regulates the expression of these proteins, thus manifesting its antiviral role [64]. In another study, Tehrani et al. examined the co-therapeutic effect of vitamin C in individuals suffering from COVID-19 with mild as well as severe symptoms. The group that received the vitamin C intervention received intravenous ascorbic acid at a dosage of 2 g every 6 h for 5 days in conjunction with the typical therapy [65]. The level of oxygen saturation considerably increased in the ascorbic acid group. In addition, the vitamin C group showed a markedly reduced respiratory rate. The computed tomography scans of the lungs of participants in both groups, as stated by radiologists, were also evaluated. After the end of the treatment, the rate of lung involvement in the vitamin C group was much more lower compared to the control group, as reported by the radiologists [65]. Furthermore, the study by Tosato et al. combined the use of l-arginine and ascorbic acid administered orally twice daily and demonstrated improved performance in walking, muscle force, endothelium function, and fatigue in adults presenting long COVID-19 [66]. One study looking at the impact of elevated intake of ascorbic acid (intravenous) showed that the control group receiving vitamin C stayed in hospital less (about two days less) than the group receiving only the basic treatment [67].

In addition, Fogleman et al. suggested that taking nutritional supplements may improve the symptoms and quality of life of individuals presenting minor to medium COVID-19 infection [68]. However, comparing vitamin C, melatonin, and placebo, they did not find any difference in the group taking placebo and the group consuming ascorbic acid, while they found improvement in the group receiving melatonin. Specifically, vitamin C administration (1000 mg orally for 14 days) had no effect on disease progression [68]. Additionally, a retrospective study examining self-reported frequent nutritional supplement use data from 372,720 UK participants in the initial waves of the pandemic up to 31 July 2020 was performed [38]. In general, this study showed that ascorbic acid supplementation did not exert any considerable impact against SARS-CoV-2 infection. A beneficial relationship was only found concerning male participants aged >60 years receiving vitamin C supplements in testing positive for SARS-CoV-2 [38]. Accordingly, a randomized clinical trial evaluated whether high-dose ascorbic acid may decrease the symptoms’ intensity or duration in 214 individuals presenting SARS-CoV-2 infection [26]. Again, vitamin C supplementation did not significantly lower symptoms’ severity and duration compared with standard care [26]. Moreover, a two-center, non-interventional, retrospective cohort study conducted on 296 patients admitted to an intensive care unit (ICU) with a proven COVID-19 infection investigated the potential effect of a low dosage of ascorbic acid as an adjunctive treatment in COVID-19 infection disease [39]. Once more, a low dosage of ascorbic acid as an adjunctive treatment against COVID-19 was not related to death rates benefits; however, it was associated with a reduced prevalence of thrombosis [39]. Thus, there is insufficient evidence thus far to recommend ascorbic acid supplementation for preventing or treating COVID-19 pathology.

#### 3.1.5. Vitamin D

Vitamin D constitutes a secosteroid hormone with a recommended daily intake of 5 μg, which could minimize COVID-19 negative outcomes by modulating either the renin-angiotensin system (RAS), and the innate and adaptive cellular immunity, or the physical barriers, and the host frailty and comorbidities [69]. Vitamin D belongs to the class of fat-soluble hormones and is currently known as a steroid hormone. Two types of vitamin D have been identified: D2 (ergocalciferol) derived mainly from plant sources and D3 (cholecalciferol) derived mainly from animal sources [40]. With no effective SARS-CoV-2 treatment, chemoprevention, or immunization, concentrating on the urgent repurposing of current medications offers promise against the COVID-19 pandemic. A novel unbiased genomics-guided tracking of SARS-CoV-2 targets in human cells recognized vitamin D as one of the top three compounds exhibiting putative infection modification patterns. This premise is supported by increasing pre-clinical and epidemiologic observational evidence. In support of this view, Annweiler et al. anticipated that vitamin D administration might enhance COVID-19 prognosis [40]. 

Vitamin D is considered as an immunoregulatory hormone with enhanced effectiveness against upper pulmonary tract infections. Its immunomodulatory activity is directly related to its anti-inflammatory activity. Vitamin D is involved in T-lymphocyte maturation and can divert helper type 17 (Th17) cells to anti-inflammatory, regulatory T cells (T-reg) [40]. The proinflammatory cytokines IL-1, IL-6, IL-12, and TNF-α seem to be decreased by this differentiation, whereas the anti-inflammatory IL-10 is increased. Additionally, vitamin D affects the transition of T helper cells from type 1 to type 2 [40]. As a result of this migration, pro-inflammatory cytokines are also present in lower quantities. Inhibiting NF-κβ, which is likewise a function of vitamin D, can also reduce these cytokines. As a result, vitamin D can decrease the cytokine cascade associated with COVID-19 through multiple mechanisms. Vitamin D is recommended for use against COVID-19, since it has been demonstrated to lower angiotensin-converting enzyme 2 (ACE2) receptor levels and T cell differentiation. In this aspect, a multicenter, randomized controlled clinical trial in individuals aged ≥65 years and presenting COVID-19 showed that an elevated dosage of vitamin D supplementation could be considered as an efficient, well-tolerated, and simply and directly available co-treatment for COVID-19, the prevalence of which can be increased considerably [41].

A multicenter randomized clinical trial was performed to compare the impacts of 5000 IU daily oral vitamin D_3_ supplementation compared to 1000 IU daily oral vitamin D_3_ supplementation on symptoms’ and other clinical parameters’ recovery in mild to moderate COVID-19 patients presenting suboptimal vitamin D levels [41]. This study found that a lack of vitamin D could be linked to an elevated probability of COVID-19 severity [41]. In Tan’s et al. study, it was found that in older individuals with COVID-19, a vitamin D/Mg/vitamin B12 mixture was associated with a considerable reduction in the percentage of patients presenting clinical deterioration and requiring oxygen support, intensive care support, or both of them [42]. This research suggests that population-based randomized controlled clinical trials should be conducted to determine the overall impact of this mixture in reducing the symptoms’ intensity of COVID-19 [42]. In this aspect, AlSafar et al. found that inadequate blood concentrations of the neurohormone vitamin D are linked to an enhanced probability of symptoms’ intensity and death rates from COVID-19. In spite of the worldwide immunization deployment and good first outcomes, the emphasis remains on additional COVID-19 prevention measures [43]. However, vitamin D at a dosage of 800 or 3200 IU/day cannot affect the defensive effectiveness or immunogenicity of SARS-CoV-2 vaccination when administered to adults presenting lower levels of vitamin D at disease onset [44].

By collecting and scrutinizing the currently existing clinical data, seven meta-analyses and systematic reviews and three subsequent clinical trials claimed that reduced vitamin D levels enhanced the predisposition to COVID-19 and the probability of higher disorder severity and mortality [70]. Moreover, five meta-analyses and systematic reviews have currently examined vitamin D supplementation for its capacity to prevent acute pulmonary infection, as well as COVID-19 [70]. A newer randomized controlled clinical trial evaluating vitamin D supplement intervention (52,000 IU monthly) in 198 patients with a 3- to 6-month follow-up of SARS-CoV-2 infections was also performed [45]. The above study clearly showed that vitamin D supplementation in patients with 25(OH)D_3_ concentrations ranging from 20 to 100 ng/mL exhibited a decreased rate of SARS-CoV-2 infection than the usage of nutritional-hygienic measures at 6 months follow-up [45]. In addition, a systematic review and meta-analysis of primarily cohort surveys concluded that vitamin D supplementation could be beneficial for the decrease in COVID-19 symptoms’ intensity, including mortality [71]. In spite of the above hopeful results, it remains unclear whether vitamin D supplementation may prevent or co-treat of COVID-19 in healthy individuals. However, we cannot rule out that it could exert beneficial effects in those patients diagnosed with malnourishment, vitamin D insufficiency, or low vitamin D levels.

#### 3.1.6. Omega-3 Fatty Acids

Omega-3 fatty acids with a recommended daily intake of 1000–16,000 mg have demonstrated benefits for the immune system, also exerting strong anti-inflammatory properties [72]. Omega-3 fatty acids are well-recognized for their ability to combat viruses such as influenza [73]. The oxygenation of COVID-19 patients was shown to be enhanced by omega-3 fatty acids. However, the above evidence should be used with caution, as several items of data reveal an increase in oxidative stress and inflammation in individuals with COVID-19 [74].

Numerous scientific and clinical research studies have linked omega-3 fatty acids consumption to improvements in general health, cardiovascular health, eye health, and brain development [46]. In addition, it is believed that these fatty acids possess hepatoprotective, neuroprotective, hypolipidemic, and anti-inflammation, as well as antioxidant, activities [75]. Several of these research studies explain the immunomodulatory impacts of omega-3 fatty acids and their power to increase and regulate mood, suggesting that omega-3 fatty acids could be capable of boosting mental health, while simultaneously enhancing human immunity against COVID-19 [47,75,76].

Sedighiyan et al. performed a blinded, randomized, controlled clinical trial in a Tehran hospital to determine whether omega-3 fatty acids may be a suitable adjunct to other anti-inflammatory drugs for COVID-19 patients (Amir-Alam) [46]. Thirty-three individuals with COVID-19 were allocated into two groups; one received hydroxychloroquine as a placebo and the other received hydroxychloroquine along with 2 g of each omega-3 fatty acid for two weeks [46]. Compared to the control group, omega-3-supplemented patients showed a considerable improvement in all clinical symptoms except for bodily pain, tiredness, appetite, and olfactory symptoms. Both c-reactive protein (CRP) and the erythrocyte sedimentation rate (ESR) were also reduced by omega-3 supplementation in comparison with the control group following therapy. Nevertheless, after supplementation, no variations in liver enzyme serum concentrations between groups were noted [46]. Moreover, a strong disadvantage of the above study was the small number of the enrolled individuals.

Several clinical symptoms, including body discomfort, fatigue, and a loss of appetite, improved in the group receiving omega-3 fatty acid supplements, according to the existing findings. The loss of smell did not improve, and there was no difference between groups in the concentration of liver enzymes. According to this study [76], a moderate dosage of omega-3 fatty acids may hold promise for reducing COVID-19-induced inflammation-related clinical symptoms [76].

Doaei et al. [47] also revealed that the interventional group exhibited a significantly greater one-month survival rate, arterial pH, HCO_3_, and base excess (Be) level, as well as arterial blood urea nitrogen (BUN), creatinine (Cr), and potassium (K) level, than the control group. The blood glucose, sodium (Na), hematocrit test (Hct), Ca, phosphorus (P), mean arterial pressure (MAP), O_2_sat, PO_2_, PCO_2_, white blood cells (WBCs), Glasgow Coma Scale (GCS), hemoglobin (Hb), platelet (Plt), partial thromboplastin time (PTT), and albumin levels were not substantially diverse between the two groups [47]. Due to the beneficial effects of omega-3 dietary supplementation on acidosis and renal function, the results of this randomized clinical study suggest that it may improve clinical outcomes in COVID-19-infected individuals. Hence, it is crucial to perform additional clinical investigations, utilizing a variety of omega-3 polyunsaturated fatty acids (PUFAs) dosages, larger sample sizes, and longer time periods [47].

#### 3.1.7. Glutamine

Glutamine, with a recommended daily intake of 3–6 g, exerts a crucial impact on several functions of the immunological system. It is a vital source of energy for immunological system cells, such as white blood cells (WBCs) and specific intestinal cells. Additionally, glutamine deficiency can impair the immune system’s ability to function. After severe injuries such as burns, high-protein high-glutamine diets or glutamine supplements are frequently prescribed [48]. Glutamine supplements may also improve human health, reducing infections and shortening hospital stays after surgery. In other words, glutamine may significantly contribute to immune function. Nonetheless, during illness or injury, the body may be unable to produce sufficient glutamine amounts. Glutamine supplements can enhance immune function and preserve the body’s protein stores [48].

Glutamine supplement treatment for a period of 5 days also considerably enhanced patients’ appetite relative to the control group, and four days of glutamine supplementation significantly reduced serum levels of TNF-a, high-sensitivity CRP, and IL-1 [48]. In the above study, patients who agreed to take glutamine comprised the interventional group, which was distinguished from patients in the control group who did not take glutamine [61]. The 230 individuals with COVID-19 in the control group were comparable to the 228 individuals with COVID-19 who used l-glutamine in terms of age, gender, and clinical condition [48]. The interventional group received 10 gr of glutamine three times daily for a period of 5 days. At the last day of the intervention, additional blood samples were received to measure serum concentrations of IL-1, TNF-a, malondialdehyde, and total antioxidant capacity [48].

A decrease in appetite causes a deficiency in protein, zinc, vitamin C, and other micronutrients that boost immunity, which exacerbates the disease’s effects. In comparison to the control group, glutamine supplementation significantly enhanced hunger levels throughout the course of five days in the study’s experimental group. Both IL-1 and TNF-a levels in the blood were considerably reduced after 4 days of glutamine supplementation, according to the findings of this study [48]. Moreover, individuals with COVID-19 have higher oxidative stress amounts than healthy people. By ingesting 10 g of glutamine three times daily, COVID-19 patients presenting pulmonary infections can reduce their concentrations of IL-6, TNF-a, and hs-CRP and increase their appetite. Thus, this supplement can aid these patients in avoiding malnutrition by stimulating their appetite [48].

#### 3.1.8. Resveratrol

Resveratrol with a recommended daily intake of 50–500 mg belongs to the polyphenols, which have considerable antioxidant properties. It is produced by plants—as with other phytochemicals—in response to pests and UV radiation. In small quantities, it is known to be found in about 70 plant species, mainly in the skin of the fruit. As an antioxidant, it can also reduce the oxidation of ‘bad’ LDL cholesterol, which causes atherosclerosis [49]. Resveratrol, in addition to its strong antioxidant property, exerts a beneficial effect on the onset and progression of atherosclerosis, prevents oxidative stress and the formation of reactive oxygen radicals, exerts anti-inflammatory action, inhibits the modification/oxidation of LDL and platelet aggregation, modifies the complications of atherosclerosis, and exerts cancer-preventive properties [49].

In addition, according to Marlies de Ligt et al., the cell-surface receptor that allows SARS-CoV-2 to go into the cells is ACE2 [49]. Due to the high expression of ACE2 and the resulting increased viral transmission in obese individuals with COVID-19, adipose tissue (AT) is a possible SARS-CoV-2 reservoir. Despite the lack of human investigations, rodent and cell research studies have shown that the polyphenol resveratrol changes ACE2. Marlies de Ligt et al. [49] examined whether resveratrol supplementation for a period of 30 days in obese persons could alter renin-angiotensin system (RAS) components in both AT and skeletal muscle. This study was a placebo-controlled cross-over clinical trial. Resveratrol showed a low impact on the expression of angiotensinogen, ACE2, and angiotensin II type 1 receptor (AT1R) in AT or skeletal muscle RAS components, but it considerably decreased ACE2 (by about 40%) and leptin (by about 30%). The above results support evidence that resveratrol treatment decreases ACE2 in AT, which may inhibit the spread of SARS-CoV-2 in COVID-19 [49].

Marlies de Ligt et al. also found that resveratrol treatment significantly reduced the gene expression of leptin and ACE2 in central and peripheral AT in comparison with a placebo [49]. There were no discernible differences in the expression of the AT genes for AGT, ACE2, and AT1R between the resveratrol group and the control group. Moreover, the expression of the angiotensinogen (AGT) and ACE2 genes in skeletal muscle did not differ significantly from a placebo [77]. The above findings showed that resveratrol administration substantially lowers ACE2 and leptin expression in AT. These results may have ramifications concerning obese people, since resveratrol supplementation may reduce their probability of contracting SARS-CoV-2 by decreasing the expression of the ACE2 receptor in AT. Additionally, resveratrol reduced AT leptin expression, which may exert favorable impacts on the outcome of COVID-19. It should be emphasized that after SARSCoV-2 entrance, ACE2 expression is inhibited, which could lead to higher angiotensin II levels and poor clinical outcomes in individuals with COVID-19 [77]. Moreover, the COVID-19 pandemic resulted in unhealthy lifestyles, namely a rise in overweight and obesity, which has been associated with an elevated chance of poor COVID-19 outcomes. In this aspect, web-based health services might be proven beneficial, particularly in times of severe constraints [77].

#### 3.1.9. Beta-Glucans

Beta glucans are polysaccharides with a recommended daily intake of 200 mg, which are considered as nature’s immunological regulator, reinforcing prime immune cells and enhancing and balancing optimal natural immunity. The beta-1,3-D glucans in cereals help to control glucose and cholesterol levels, enhancing weight loss while protecting the gastrointestinal system. Similarly, the beta-1,6-D glucans in mushrooms shield the immune system. In general, they are considered “functional ingredients” since their regular consumption has been found to exert a beneficial impact on human health [50].

Furthermore, 72 volunteers participated in a single-center, randomized clinical trial, where they were either given a placebo or a coactive mixture of yeast-based components with a special 1.3/1.6-glucan complex and a group of heat-treated probiotic Saccharomyces cerevisiae enriched with Se and Zn (ABB C1^®^) the day after receiving an anti-inflammatory medication, according to Rodriguez et al. [50]. For the influenza vaccination group, the treatment plan lasted 30 days, while for the COVID-19 vaccine group, it lasted 35 days. Following the second dosage of the COVID-19 immunization, the mean CD4 + T cell counts in the ABB C1^®^ group were elevated from 910.7 cells/L at baseline to 1000.2 cells/L, but they fell in the placebo group from 1055.1 cells/L to 929.8 cells/L [50]. Both CD3 + T and CD8 + T cells underwent the same modifications. In the COVID-19 cohort, the ABB C1^®^ supplement group experienced higher elevations in IgG and IgM than the placebo group. Patients receiving the active medicine experienced higher serum levels of Zn and Se than those receiving a placebo. ABB C1^®^-related significant adverse events or problems with tolerance were not recorded. Thus, this study’s findings support ABB C1^®^’s ability to stimulate trained immunity [50].

Originally intended to have 45 participants in each cohort, recruitment issues led to the inclusion of 72 participants (27 men and 45 women), 29 of whom (40.2%) were above the age of 70 [50]. The influenza vaccine cohort had evaluations at three different times: baseline, 7th day (middle assessment), and 30th day (ultimate assessment). Concerning COVID-19 vaccine cohort, evaluations were carried out at baseline, on Days 7, 21, and 35. ABB C1^®^ (*n* = 33) or a placebo (*n* = 34) were administered to 67 out of the total 72 subjects [50]. The remaining five subjects violated the protocol by not receiving a vaccination, so they were dropped from the analysis. In total, 32 vaccinated participants received a supplement with ABB C1^®^ (COVID-19, *n* = 18; influenza, *n* = 14) and 32 received a placebo (COVID-19, *n* = 14; influenza, *n* = 14) among the vaccinated participants who completed the clinical trial [50]. Subjects who received the COVID-19 mRNA vaccine from Pfizer-BioNTech, supplementation with ABB C1^®^, a consortium of S. cerevisiae-glucan complex, and were supplemented with Se and Zn exhibited T-cell responses after a single dosage [50]. Compared to the placebo group, the ABB C1^®^-supplemented group showed higher concentrations of cellular and humoral immune responses, which were not affected by participants’ age or gender. According to this recent study, antibody markers showed a clear association with vaccine effectiveness, as well as a high negative association with COVID-19 risk [50].

#### 3.1.10. Probiotics

Probiotics with a recommended daily intake of 10^9^–10^10^ colony forming units (CFUs) are tiny microorganisms that deliver several health benefits to their host. The major place of action for probiotics is the large intestine. Hence, a probiotic must avoid the negative impacts of stomach acid and the relevant digestive enzymes to access the large intestine. Once in place, probiotics provide their purported benefits by promoting the growing and metabolic activities of the bacteria that ferment them [78]. The main general target bacteria for probiotic activity are the biphospho-bacteria and lactobacilli, although this may change as knowledge of microbial diversity and functionality expands [79]. It cannot, however, be excluded that probiotics have a direct effect on human health, e.g., through immune system mechanisms [79]. 

Several studies have examined COVID-19 patients’ gut microbiotas; however, nothing is known about the dynamics of the resistome, a gene bank for antimicrobial resistance (ARGs). The effects of antibiotics or probiotics on the ARGs reservoir were shown in the study by Qi Su et al. (2022), which examined the potency of the resistome from the time of diagnosis to 6 months following viral clearance [51]. They profiled the longitudinal fecal metagenomes of 142 individuals with COVID-19 infection. At baseline, antibiotic-naive COVID-19 patients had ARGs that were more abundant, diversified, and ubiquitous than non-COVID-19 controls. Tetracycline, vancomycin, and multidrug-resistant genes were the primary drivers of resistome-expansion for at least 6 months next to SARS-CoV-2 elimination [51]. Those presenting larger resistomes were more susceptible to the post-acute COVID-19 syndrome and Klebsiella sp. Throughout the acute infection and recovery stage of COVID-19, oral probiotics (synbiotic formula, SIM01) considerably lowered the ARGs reservoir in the intestinal microbiome. On the other hand, antibiosis treatment increased the amount of ARGs. Overall, these findings provide new light on the potency of the ARGs reservoir in COVID-19 patients and promote the expectancy that microbiome-facilitated treatment may support patients with COVID-19 that have accumulated ARGs, decreasing their burden [51].

Furthermore, between 1 March 2020 and 31 January 2021, 24 patients who had already received treatment and 66 individuals presenting proven SARS-CoV-2 infection were assigned [51]. They were monitored for as long as 9 months after the virus was contained. Before and after contracting SARS-CoV-2, each of these individuals went at least 6 months without antibiotic therapy. Also, 66 non-COVID-19 individuals with the same age, sex, and comorbidities as the individuals with COVID-19 were enrolled [51]. Using metagenomic profiling, the resistome of a feces sample was analyzed. Using Bray–Curtis dissimilarities, these researchers were able to distinguish between the resistomes of baseline samples from individuals with COVID-19 and without COVID-19. In samples of individuals with COVID-19, the diversity and number of discovered ARGs were significantly higher. By normalizing to the overall number of 16S reads, the total abundance of ARGs in samples from individuals with COVID-19 remained significantly higher compared with the samples from individuals without COVID-19 [51].

Knowing what affects the human intestinal resistome, as well as how to inhibit resistome proliferation, means fighting AMR is easier [51]. This study uses information from shotgun metagenomic sequencing of samples from various human populations to show whether probiotics and antibiotics could affect the resistome. When COVID-19 was admitted, they observed a sizeable development of the resistome, which continued to grow throughout the virus-positive interval and remained for months after virus clearance [51]. Although using antibiotics, the virus was present and additional aided in the development of the resistome, taking an over-the-counter probiotic (Synbiotic Formula, SIM01) prior to or after virus clearance, which was related with a decrease in the amount and abundance of ARGs accumulated in individuals with COVID-19. Moreover, no rebound effect was noted throughout the 12-week follow-up [51].

A randomized, multicenter clinical trial has recently been performed in 200 patients with a complaint of post-COVID-19 fatigue [52]. The interventional group (*n* = 100) consumed the oral probiotic supplements for 14 days and the non-interventional group (*n* = 100) consumed a placebo. The probiotics’ supplemental intervention led to the resolution of fatigue in a higher proportion of patients of the interventional group than the non-interventional group on 14th day. Patients in the interventional group also exhibited a substantially higher decrease in the overall, as well as the physical and mental fatigue, scores at all time points compared to the non-interventional group [52]. Collectively, the currently available evidence concerning the impacts of probiotics’ supplementation on preventing COVID-19 infection and disease symptoms’ severity seem promising but not quite adequate. Further population-based clinical studies should be performed to derive conclusive results.

### 3.2. SARS-CoV-2 and Dietary Patterns

Se, vitamin C, and vitamin D deficiency are representative examples of micronutrient deficits associated with SARS-CoV-2 infection and modest results in observational or ecological studies currently exist. According to a small number of clinical studies, individuals presenting SARS-CoV-2 infections who received vitamin C and D supplements had greater survival rates and less severe sickness [80]. As a result of the psychological consequences and limitations forced by the lockdown, the pandemic itself resulted in deteriorating lifestyle patterns in Italy. In this aspect, there was an increase in the consumption of unhealthy foods and a decrease in both Mediterranean diet (MD) compliance and physical activity [80]. The MD is a plant-based diet, including a high intake of fruits, vegetables, cereals, legumes, tree nuts, seeds, and olives. Olive oil is the major source of added fat and is consumed along with an increased moderate intake of fish and seafood (approximately two servings weekly), moderate per week consumption of eggs, poultry, and dairy products (two servings weekly), a low intake of red meat (approximately a maximum of two servings weekly), and processed meat (approximately one serving weekly) [53,54,81,82].

The above combination of eating behaviors includes enhanced consumptions of nutrients with anti-inflammatory, antioxidant, and immunomodulatory properties. These nutrients contain dietary fiber, unsaturated fats, PUFAs, vitamins, minerals, and diverse beneficial phytochemical compounds like polyphenols, triterpenoids and flavonoids. Notably, an inverse relationship between MD adherence and the risk of cancer, cardiovascular diseases, neurodegenerative disorders, and metabolic disorders has been supported by various previous meta-analyses [53,54,81,82]. Moreover, MD can decrease the risk of sepsis and respiratory infections, and inflammation, as implied by a decrease in CRP and proinflammatory cytokines. Contrary to the incidence of SARS-CoV-2, the trend of MD adherence in Italy is increasing from the North to the South [53]. The MD has been considered as a sufficient approach for fighting SARS-CoV-2 infection and minimizing severity-associated concerns like obesity, diabetes, and cardiovascular diseases. To the best of our knowledge, no study has thus far explicitly investigated the relationship of MD compliance with the prevalence of SARS-CoV-2 pathology [53,54,81,82]. Healthcare professionals (HCPs) are mostly susceptible to SARS-CoV-2 infection because of the nature of their job. Medical staff were the focus of 126,394 cases, or 3.8% of all infections in Italy. Notably, 10% of HCPs are expected to be diagnosed with SARS-CoV-2 infection [53,54,81,82].

According to Ponzo et al. [53], those diagnosed with SARS-CoV-2 illness (of any seriousness) had a considerably elevated consumption of saturated fats and proteins and decreased intake of carbohydrates and fibers. The MD compliance to consume fruits, vegetables, grains, and olive oil were much lower among the former. When scores were examined as categorical variables, the outcomes were similar [53]. Each additional MD point was related with a 12% decrease in the chance of contracting SARS-CoV-2. The likelihood of infection was inversely correlated with grain consumption in the model that included each individual MD component. If the scores were seen as categorical factors, the outcomes did not substantially alter [53].

In addition, asymptomatic SARS-CoV-2 carriers had lower intakes of saturated fat and were much younger than symptomatic carriers. The limited number of HCPs who were admitted to the hospital were notably older, had noticeably worse food habits, and had a lower health status compared to asymptomatic and symptomatic patients [53,54,81,82]. The significant variables were then included in a logistic regression model through a stepwise backward selection approach. The connection between age and the percentage of saturated fats and the severity of the infection remained statistically significant. This result persisted regardless of whether the MD components were added as continuous or categorical variables to the model’s initial variables [53,54,81,82].

Moreover, a prospective cohort of 9677 middle-aged university graduates in Spain demonstrated that individuals presenting moderate compliance to the MD exhibited a considerably lower risk of COVID-19 infection [54]. Additionally, those participants presenting a higher MD compliance showed the lowest probability as compared with participants with a low MD compliance. This inverse relation persisted as robust within subgroups, as well as in sensitivity analyses [54]. A more recent cross-sectional survey including 3721 adults was performed to assess the possible relation of MD compliance with several sociodemographic, anthropometric, and lifestyle factors during the COVID-19 pandemic period [55]. The above study showed that a higher MD adherence was independently associated with a lower probability of central obesity, enhanced physical activity, higher sleep quality, improved quality of life, and lower risk of anxiety and depression throughout the COVID-19 pandemic [81]. Moreover, a systematic review including 13 observational studies has recently been performed, revealing that healthy dietary patterns such as the MD may decrease the probability of SARS-CoV-2 infection or COVID-19 [54]. Notably, higher dietary intakes of fruits, vegetables, and fibers have been related with a reduced likelihood of COVID-19 infection or developing severe COVID-19 symptoms [54].

Furthermore, intermittent fasting and a ketogenic diet may be proved as other fascinating nutritional approaches with potential beneficial impacts on both COVID-19 and PASC. These dietary patterns have been found to exert positive impacts on metabolic health and obesity. Thus, taking into consideration the fact that diminished metabolic health is involved in the growing severity of both COVID-19 and PASC symptomatology, it could be interesting to evaluate the effectiveness of both dietary patterns by performing relevant clinical trials in the near future.

## 4. Discussion

A focus on immune system enhancement shows promising preliminary evidence against COVID-19. Since healthy nutrition is inextricably linked to a person’s health, achieving optimal immune responses is a crucial issue. Specifically, dietary supplements provide micronutrients (vitamins, minerals) that are typically deficient in the average diet. Moreover, it has been documented that some micronutrients contain immunomodulatory effects that can be used to prevent disease or boost the immune system when it is already compromised. Vitamins such as vitamin D, vitamin C, etc., and trace minerals such as Zn, Se, etc., have been proven to reinforce the clinical picture of individuals with COVID-19 [83].

Specific quantities of each nutrient are required for the body to function properly. However, no food includes all the essential elements, and the combination of foods cannot always ensure an appropriate intake. Therefore, they must be taken exogenously in the form of dietary supplements. More to the point, their use is highly recommended for vegetarians and vegans, as dietary restrictions sometimes lead to severe deficits in these population groups. Fernandes et al. [84] performed a study comprised 200 patients with a mean age of 55.5 ± 14.3 years and a BMI of 32.2 ± 7.1 kg/m^2^, 109 (54.5%) of whom were male. Although the difference between the groups at postintervention after Bonferroni’s correction was not noteworthy, granulocyte-macrophage colony-stimulating factor (GM-CSF) levels showed a considerable group-by-time interaction impact. The remaining outcomes did not show any meaningful influence [84]. People attempting to lose body weight have a similar pattern. Dietary supplements are utilized for body weight loss and because some of them improve satiety. In addition, the usage of nutritional supplements during pregnancy has been established to ensure that all nutrients necessary for fetal development are delivered. Similar to women (pregnant or lactating), young children, the elderly, and athletes have increased vitamins and minerals’ requirements. Additionally, they are used to activate the immunological system [77]. Vitamin C has been shown to be useful to people suffering from COVID-19 sickness in 2019. The purpose of the above research focused on the evaluation of the effect of vitamin C supplementation on pathological biomarkers and life length in severely sick COVID-19 patients [85].

Deficiencies in specific nutrients like vitamin C, vitamin D, and omega-3 fatty acids may enhance the risk of infections. Hence, there is an increasing focus on immunological system-improving supplements, which may contribute to the prevention of COVID-19 or to attenuate its symptomatology. Several substances have not completely been evaluated for this disorder thus far. Nevertheless, it is expected that they could promote the prevention or the reduction in the symptoms of the common cold, influenza, and other pulmonary disorders. Thus, several researchers believe that they could also be efficient against COVID-19 infection [37,86,87].

Vitamin C contributes significantly to immunity because of its antioxidant, antibacterial, and antiviral activities, as well as its action on immunological regulators. Its insufficiency compromises immunological function and heightens infection vulnerability. The impacts of vitamin C supplementation on the immunological system are dependent on the individual’s vitamin C levels, according to existing research. Conventional vitamin C consumption has been shown to decrease the duration and severity of the common cold and the probability of colds in persons exposed to considerable physical stress. It is also favorable for those presenting pneumonia who have low vitamin C levels, and those presenting virus diseases, such as shingles. Vitamin C antioxidant actions could also reduce oxidative stress in the case of infections [54]. According to Ghelani’s et al. research [88], vitamin D shortage and insufficiency are very common in the global population. Remarkably, researchers have found a relation between COVID-19 symptoms’ intensity and vitamin D levels in the previous 2 years [88].

The COVID-19 epidemic has caused physical, economic, and psychological disruptions on a global scale [89]. Countries were compelled to impose strict health laws and social segregation practices to arrest the spread of the illness. The social confinement order, teleworking, and the shutdown of schools and businesses seem to have exerted a significant negative influence on individuals psyches and daily habits, especially in countries that have suffered from prolonged containment measures [89]. The containment effort has focused special attention to the immunological system’s function and its relationship with diet to create a stronger functional response and more efficient protection against SARS-CoV-2.

Adequate nutrition appears to provide some resistance to the virus and has a significant impact on infection management [90], the response to treatment, and long-term consequences of infection [86]. When fighting against an infection, the immune system must put in a lot more time and effort than normal. As a result of the increased demand for energy, biosynthetic substrates, and regulatory chemicals, the metabolic rate increases in tandem with the increasing activity. Eating a well-rounded, nutritious diet may help to strengthen the immunological system, which is crucial for warding off illness. However, a worse diet has the opposite impact, causing inflammation and triggering oxidative stress that might compromise the immune system and ultimately alter the result [91]. According to most studies, a major portion of the adult population’s eating habits are harmful to their health. Every day, individuals should consume a range of nutrients, as suggested by WHO and national health care institutes. Dietary supplements, based on the European Food Safety Authority, are meant to address dietary insufficiency, retain appropriate consumption of specific nutrients, or support certain physiological processes [92].

There was an initial emphasis on stopping the spread of the coronavirus illness (COVID-19) worldwide. The current health condition of COVID-19 survivors is an obvious priority for medical practitioners. Indeed, recent research has uncovered post-COVID-19 syndrome, which includes a lack of food intake, a decline in lean body composition, and mild inflammation. Persistent functional impairment (such as fatigue and muscular weakness, dysphagia, appetite decline, and taste/smell abnormalities) and psychological anguish might further complicate the rehabilitation process [93]. For this reason, one of the cornerstones of caring for these patients is to perform a thorough assessment of nutritional status (including food intake, anthropometrics, and body composition) [94]. Alternatively, personalized food suggestions are the most effective method for promoting human health and a speedy comeback. To help nutritionists, customized dietary therapies are needed for patients recovering from COVID-19 infection, which may be produced by compiling research on the function of nutrients and dietary supplements in post-COVID-19 complications.

## 5. Conclusions

Several dietary supplements can exert promising effects for preventing and co-treating COVID-19 infection disease and its complications. However, the currently available clinical studies in humans remain limited, reinforcing the strong demand for further research to derive conclusive results. As the COVID-19 infection disease persists until today, any research for its management and monitoring is highly recommended. An urgent concern beyond preventing and treating COVID-19 infection disease deals with its long-term negative effects and whether receiving specific dietary supplements and/or adopting a healthy dietary pattern may attenuate or even minimize their prevalence.

## Data Availability

Not applicable.
